# Structure and Function of the Bestrophin family of calcium-activated chloride channels

**DOI:** 10.1080/19336950.2021.1981625

**Published:** 2021-10-06

**Authors:** Aaron P. Owji, Alec Kittredge, Yu Zhang, Tingting Yang

**Affiliations:** a Department of Pharmacology, Columbia University, NY, USA; b Department of Ophthalmology, Columbia University, NY, USA

**Keywords:** Bestrophins, Best1, Best2, bestrophin structure, Bestrophin function

## Abstract

Bestrophins are a family of calcium-activated chloride channels (CaCCs) with relevance to human physiology and a myriad of eye diseases termed “bestrophinopathies”. Since the identification of bestrophins as CaCCs nearly two decades ago, extensive studies from electrophysiological and structural biology perspectives have sought to define their key channel features including calcium sensing, gating, inactivation, and anion selectivity. The initial X-ray crystallography studies on the prokaryotic homolog of Best1, *Klebsiella pneumoniae* (KpBest), and the Best1 homolog from *Gallus gallus* (chicken Best1, cBest1), laid the foundational groundwork for establishing the architecture of Best1. Recent progress utilizing single-particle cryogenic electron microscopy has further elucidated the molecular mechanism of gating in cBest1 and, separately, the structure of Best2 from *Bos taurus* (bovine Best2, bBest2). Meanwhile, whole-cell patch clamp, planar lipid bilayer, and other electrophysiologic analyses using these models as well as the human Best1 (hBest1) have provided ample evidence describing the functional properties of the bestrophin channels. This review seeks to consolidate these structural and functional results to paint a broad picture of the underlying mechanisms comprising the bestrophin family’s structure-function relationship.

## Introduction

Chloride (Cl^−^) is the most abundant anion in many biological systems and plays an essential role in various physiological processes. One of the primary roles of Cl^−^ is that of counterion to the movement of major biological cations, including hydrogen (H^+^), sodium (Na^+^), potassium (K^+^), and calcium (Ca^2+^). In this role, Cl^−^ is essential to establish the membrane potential. The movement of Cl^−^ ions across the lipid bilayer is tightly controlled by specialized channels and transporters. Cl^−^ channels, which open to allow the flow of Cl^−^ along its electrochemical gradient, can be classified as ligand-gated anion channels (including the glycine receptor and GABA_A receptor), Cystic Fibrosis Transmembrane Conductance Regulator (CFTR), CLC channels, bestrophins, and anoctamins (TMEM16 family), each of which have diverse functional properties.

Bestrophins are a family of Ca^2+^ -activated Cl^−^ channels (CaCCs) with important biomedical relevance in human eyes. Four bestrophin paralogs, Best1-4, have been identified in eukaryotes, all of which act as CaCCs upon heterologous expression [1,2–8]. Recent work into the bestrophin channels has revealed significant insight into their structural and functional mechanisms. The goal of this review article is to provide a comprehensive overview of the bestrophin family of CaCCs with an emphasis on their structure–function relationship.

## Discovery of Best1 and its relevance to eye disease

The *BEST1* gene, also known as *VMD2*, which encodes the Best1 protein in humans, was first identified through genetic linkage experiments associating it with mutations that cause the eye disease Best vitelliform macular dystrophy (BVMD) [9,10]. BVMD is a disease characterized by the accumulation of the fluorescent pigment lipofuscin within the retinal pigment epithelium (RPE) and macular lesions accompanied by progressive vision loss [11–14]. Mutations in *BEST1* were later determined to be associated with an array of genetic eye disorders termed “bestrophinopathies,” including BVMD, adult-onset vitelliform macular dystrophy (AVMD), autosomal recessive bestrophinopathy (ARB), autosomal dominant vitreoretinochoroidopathy (ADVIRC) [15,16], and retinitis pigmentosa (RP) [17,18]. The clinical manifestations of bestrophinopathies and the role of Best1 in these diseases are reviewed elsewhere [13,19,20]. Best1 is predominantly expressed in the RPE of the human retina, but it is not exactly clear how mutations in *BEST1* lead to the molecular pathology of bestrophinopathies [20–23].

The RPE forms the outer blood-retina barrier and plays a crucial role in maintaining retinal physiology through the transcellular transport of water, ions, metabolites, nutrients, and waste products [24]. The transepithelial potential of the RPE is created by a difference in the resting potential between its basolateral and apical membranes and these different resting potentials are the result of the localized distribution and specific activation of particular ion channels and transport proteins [25,26]. The depolarization of the RPE basolateral plasma membrane, where Best1 is expressed, changes the trans-tissue potential and leads to the light peak (LP) current, measurable in the clinical setting by an electrooculogram (EOG) [27–30]. Bestrophinopathy patients commonly display a reduced LP, which is believed to represent a Ca^2+^-dependent Cl^−^ current at the basolateral plasma membrane of RPE. As Best1 is the right type of ion channel at the right location, and indeed is indispensable for this current [31], it is the primary candidate that generates the LP. In addition to this role, Best1 is also suggested to regulate voltage-dependent Ca^2+^ channels and to contribute to volume regulation [32–36].

## Expression and function of bestrophins

In addition to the RPE, Best1 expression has also been observed in mouse and human airways, the colon, kidneys, parts of the central nervous system, in colonic cancer cells, and in a human pancreatic duct cell line [37–44]. Interestingly, *BEST1*-knockout mice do not exhibit aberrant RPE function, yet exhibit a severe subfertility phenotype that is consistent with high Best1 expression in mouse testis [34,45].

Following the identification of the *BEST1* locus, three other related genes were quickly discovered [46]. The three transcribed proteins were confirmed to be paralogs of Best1, and are now referred to as Best2, Best3, and Best4 [7,8]. Each protein has a unique expression pattern, highlighting the bestrophin family’s evolution to perform similar yet specific functions throughout the body.

Best2 is expressed in the basolateral membrane of the non-pigmented epithelium (NPE) of the ciliary body [47]. The basolateral membrane of the NPE has extensive folds and an enormous surface area-to-volume ratio, making it highly specialized for secretion of aqueous humor, one of its primary functions [48]. Mice lacking the *BEST2* gene have altered aqueous humor flow and decreased intraocular pressure (IOP), suggesting Best2 may be a potential pharmaceutical target for lowering IOP (e.g., in the treatment of glaucoma) [49,50]. In addition to the NPE, Best2 is also expressed in mouse airways, colon, secretory glands (e.g., sweat and salivary glands), and in olfactory sensory neurons [39,49,51–55]. Best2 plays a role in the regulation of bicarbonate transport in colonic goblet cells and sweat glands, has also been observed in guinea pig colon, and may be a potential diagnostic biomarker for colorectal cancer [44,55,56].

Best3 has been localized to vascular smooth muscles of humans, mice, and rats, where it regulates vascular tone, and in canine cardiomyocytes [57–60]. Best3 expression is also upregulated in hippocampal astrocytes in mouse brains after injury and in human neonatal brains with pathology, as well as in rat renal epithelial cells [61,62,63]. Expression of the Best4 paralog has been studied far less compared to Best1-3, but it has been identified in a distinct sub-population of human intestinal absorptive epithelial cells [64].

The expression pattern of bestrophins along fluid-secreting membranes suggests their involvement is important for fluid secretion, a physiological process that often relies on anion channels. For example, Best1 expression in airway epithelial cells and pancreatic duct epithelial cells, and Best2 expression in non-pigmented epithelial cells and colonic goblet cells, all of which secrete mucous-laden fluid, strongly suggest a functional role in fluid secretion processes [38,39,42,47,55]. Furthermore, bestrophins have been shown to exhibit a polarized expression pattern and these secretory cell types are highly polarized to facilitate the transcellular flow of fluids and osmolytes to their final compartments.

## Phylogeny

Bestrophins have been identified in mammals, birds, bony fish, amphibians, echinoderms, insects, nematodes, and flat worms with the different paralogs likely arising from gene duplication events [23,65]. They are found in some fungi and prokaryotes (e.g., gram-negative, but not gram-positive bacteria), but not in protozoans, plants, or yeast [66]. Mammals have either three or four bestrophin paralogs. For instance, humans have all four bestrophin paralogs (hBest1-4), while mice have only three paralogs and one pseudogene [23,45]. Vertebrate bestrophins share a greater sequence identity within the N-terminal, while the C-terminal is more variable and may mediate protein–protein interactions [23,65]. A thorough phylogenetic analysis found that vertebrate bestrophins cluster in organismal groups, such that sequences within the same paralog are more similar to each other than they are to different paralogs from the same species (e.g., hBest1 is more similar to mBest1 than it is to hBest2) [65]. The four paralogs share the same hydrophobicity pattern predicting four TM domains within the conserved N-terminal region, suggesting that they share a similar architecture and membrane topology [65]. The importance of bestrophins to physiology is underscored by their conservation across diverse kingdoms of life, and the high level of conservation within each paralog suggests that these paralogs evolved to maintain specific yet diverse functions.

## Bestrophins are Ca^2+^ -activated anion channels

The bestrophins residing in the plasma membrane respond to intracellular [Ca^2+^] in the low-mid nanomolar range to allow the flow of monovalent anions along their electrochemical gradient through the channel and across the cell membrane. The major sources of such free intracellular Ca^2+^ are from internal Ca^2+^ stores released through slow metabotropic signaling pathways or from the activation of Ca^2+^ channels on the plasma membrane. The relative permeability of different monovalent anions through bestrophins generally follows the Eisenman “weak field strength” lyotropic series, such that the ease of permeability of an anion is inversely related to the energy required for its dehydration [67,68]. The process of dehydration is a major limiting factor for an anion passing through the channel, resulting in a relative permeability sequence on the order of SCN^−^ > NO_3_^−^ > I^−^ > Br^−^ > Cl^−^ > F^−^ [23]. Bestrophins responds to prolonged or excessive Ca^2+^ by inactivating. Patch clamp recordings of bestrophin proteins reveal a time- and calcium-dependent “rundown” effect which is alleviated by truncating the protein’s C-terminus, providing initial evidence that this effect is autoinhibitory [2]. Additionally, bestrophins are sensitive to the nonselective Cl^−^ channel inhibitors niflumic acid (NFA), 4,4ʹ-Diisothiocyano-2,2ʹ-stilbenedisulfonic Acid (DIDS), 5-nitro- (3-phenylpropylamino)-benzoic acid (NPPB), flufenamic acid (FFA), and tannic acid [3,4,34,39,42,60,69–71]. Interestingly, all human bestrophins have been shown to have high permeability to HCO_3_^−^ under physiologically relevant conditions and with relatively high permeability ratios (P_HCO3-_/P_Cl-_ = 0.44–0.69 for hBest1-4), distinguishing them from other Cl^−^ channels, which generally exhibit relatively low HCO_3_^−^ transport [5]. The Ca^2+^ -dependent properties of different bestrophins, their relative permeabilities for different anions, and the relevant experimental setup used to derive these values are described in Table 1 to demonstrate the species- and paralog-specific qualities that must be taken into account when comparing these channels.

**Table 1.** Summary of key biophysical properties of various bestrophin paralogs from different species

Species/Paralog	Selectivity	[Ca^2+^] EC_50_ or K_D_ (Method)	Hill Coefficient	Source
hBest1	Permeability: NO_3_^−^ > I^−^ > Br^−^ > Cl^−^, P_NO3_^−^/P_Cl_^−^ = 2.7 at +80 mV	EC_50_ = 141 nM at +80 mV (Overexpression in HEK293 cells and whole-cell patch clamp)	n = 3.7 (Overexpressed in HEK293 cells and whole-cell patch clamp)	[7,88]
hBest1	P_HCO3_^−^/P_Cl_^−^ = 0.44 ± 0.4			[5]
cBest1	Permeability: NO_3_^−^ > Br^−^ > Cl^−^ Permeability: SCN^−^ > I^−^ > Br^−^ > Cl^−^	EC_50_ = 17 nM ± 3 nM (Lipid bilayer containing purified cBest1 protein)		[74,78]
hBest2	P_NO3_^−/^P_Cl_^−^ = 5.8 at +80 mV			[7]
hBest2	P_HCO3_^−^/P_Cl_^−^ = 0.69 ± 0.4			[5]
mBest2	Permeability: SCN^−^ > I^−^ > Br^−^ > Cl^−^ > F^−^ Conductance: I^−^ > Br^−^ > Cl^−^ > F^−^ > SCN^−^	EC_50_ = 230 nM at +100 mV (Overexpression in HEK293 cells and whole-cell patch clamp)		[3]
mBest2	Permeability: SCN^−^ > NO_3_^−^ = I^−^ > Br^−^ > Cl^−^ Conductance: NO_3_^−^ > I^−^ > Cl^−^ ≥ Br^−^ > SCN^−^			[4]
mBest2	Permeability: I^−^ > NO_3_^−^ > Br^−^ > Cl^−^ ≫ MeS^−^ (1.8:1.4:1.2:1:0.3)	K_1/2_ = 400 nM at −50 mV (Overexpression in HEK293 cells and whole-cell patch clamp)	n = 2.2 for WT n = 2.9 for KO (Inside-out patch clamp from mouse dendritic knob olfactory sensory neuron membrane patches w/ various [Ca^2+^], held at 50 mV)	[54]
mBest2	P_HCO3_^−^/P_Cl_^−^ = 0.63 ± 0.3			[5]
xBest2a	I^−^ > Br^−^ > Cl^−^ ≫ aspartate^−^ (2.6:1.7:1:0.15)	EC_50_ = 210 nM at +100 mV (Overexpression in HEK293 and whole-cell patch clamp)		[99]
xBest2b	I^−^ > Br^−^ > Cl^−^ ≫ aspartate^−^	EC_50_ = 228 nM at +100 mV (Overexpression in HEK293 and whole-cell patch clamp)		[99]
bBest2	Permeability: SCN^−^ > I^−^ > Br^−^ > Cl^−^ Conductance: Br^−^ > I^−^ = Cl^−^ > SCN^−^	EC_50_ = 26 nM at +100 mV (Overexpression in HEK293 and whole-cell patch clamp)		[81]
mBest3	Permeability: SCN^−^ > I^−^ > Cl^−^ > gluconate^−^ (3.5:1.3:1:0.3)	K_D_ = 161.9 ± 49.2 nM at −80 mV & 174.9 ± 51.8 at +80 mV (Overexpression in TRex293 cells and whole-cell patch clamp)	n = 2.6 ± 1.5 at −80 mV & (Overexpression in TRex293 cells and whole-cell patch clamp)	[60]
hBest4	P_HCO3_^−^_/_P_Cl_^−^ = 0.65 ± 0.03	K_1/2_ = 230 nM (Overexpression in HEK293 cells and whole-cell patch clamp)	n = 0.53 (Excised patch of hBest4-transfected HEK293, Cells at free Ca^2+^ concentrations of near-zero, 300 nM, and 100 μM)	[5]

## Overall architecture

**Figure 1.**
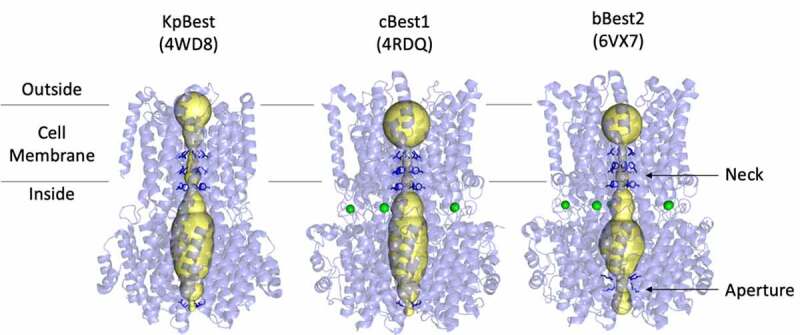
Side-by-side view of the three bestrophin homolog structures solved to date. The two major occlusions to the ion conduction pathway are labeled: the neck within the inner leaflet of the transmembrane domain, and the aperture at the end of the cytosolic domain. Amino acid side chains forming these constrictions are depicted as dark blue sticks. Ca^2+^ ions are shown in green and the ion conduction pathway at the center of the channel axis of symmetry is shown as yellow volume

The first structure of a bestrophin channel was the X-ray crystal structure of the prokaryotic bestrophin homolog from *Klebsiella pneumoniae* (KpBest, Figure 1, left), which shares 14% sequence identity with human Best1 (hBest1) [72]. The KpBest structure was determined through a structural genomics approach in which the structure of multiple prokaryotic bestrophin homologs were pursued in a high throughput manner [73]. The first eukaryotic bestrophin structure, that of chicken (*Gallus gallus*) Best1 (cBest1, Figure 1, center), which shares 74% sequence identity with hBest1, was elucidated by X-ray crystallography with the aid of a Fab monoclonal antibody fragment acting as a crystallization chaperone [74]. Both KpBest and cBest1 have been used as models to dissect molecular mechanisms of mammalian bestrophins through functional and structural studies [75–79]. Recently, the first structure of a mammalian bestrophin channel was solved by single-particle cryogenic electron microscopy (cryoEM) using bovine (*Bos taurus*) Best2 (bBest2, Figure 1, right) [80,81]. KpBest, cBest1, and bBest2 are the only bestrophin structures to date, laying the basis for structure–function studies.

The prokaryotic KpBest, avian cBest1, and mammalian bBest2 channels exhibit remarkable similarities in overall shape, dimensions, and key aspects of the ion conduction pathway. These channels comprise a homo-pentameric assembly with cyclical symmetry (C5), four transmembrane helices per protomer (20 per channel), and a flower-vase shaped ion conduction pathway traversing approximately 95 Å along the central axis of symmetry (Figure 1, yellow volumes). Bestrophins have a characteristic extra-membrane domain on the cytoplasmic side of the membrane, which encompasses a large solvent-filled vestibule through which the ion conducting pathway extends approximately 45 Å from the edge of the membrane into the cytosol (Figure 1). All three bestrophin structures exhibit two major occlusions to the ion conduction pathway along the channel axis: the “neck,” a constriction within the membrane composed of three conserved hydrophobic residues, and the “aperture,” a narrow point at the cytosolic end of the ion conducting pathway that confers paralog and species-specific channel properties. Both of these constructions have been shown to contribute to anion selectivity and gating.

## The Neck: A hydrophobic gate within the membrane

**Figure 2.**
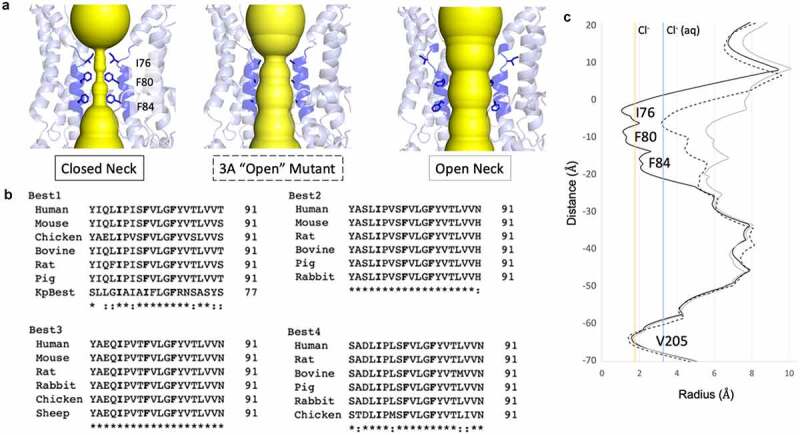
The neck is a highly conserved hydrophobic gate. A) Left, the closed neck of cBest1 has conserved I76, F80, and F84 pointing into the ion conduction pathway. Middle, crystal structure of the 3A mutant (I76A + F80A + F84A) in cBest1 has an open neck despite lack of conformational change within this pore-lining helix. Right, cryoEM structure of cBest1 with non-inactivating truncation has an open neck in which the pore-lining helix has made conformational change to widen the neck to diameter > 10 Å. B) Multiple sequence alignment across species of the four bestrophin paralogs demonstrates strict conservation of these three hydrophobic residues (bold). Note, KpBest IIF motif. Best1 sequence similarity notation does not account for KpBest. “*” denotes fully conserved residue, while “:” denotes similar residue. C) HOLE diagram demonstrates key differences in the radius of the neck constriction for the conformations depicted in A, using the same label scheme as in A. Solid black line = closed neck, dotted line = 3A mutant, solid gray line = non-inactivating mutant with open neck for cBest1. Vertical solid yellow line indicates the radius of dehydrated Cl^−^, while solid blue line indicates the radius of hydrated Cl

An ion passing through the channel from the extracellular side of the membrane will first encounter the “neck,” a hydrophobic gate located at the level of the intracellular leaflet of the plasma membrane. This channel feature is composed of three highly conserved hydrophobic residues residing on the second transmembrane alpha-helix (helix S2b, also termed transmembrane domain 2 (TMD2) in earlier studies) with the conserved hydrophobic residues pointing in toward the central axis of the channel [74,81,82,3,4,72]. In cBest1 and bBest2, the neck is composed of I76, F80, and F84 (Figure 2a, left), with their counterparts in KpBest being I62, I66, and F70, respectively. Multiple sequence alignment reveals that these three hydrophobic residues are fully conserved in eukaryotic bestrophins (Figure 2b). Early functional studies identified TMD2 as a determinant of anion selectivity in bestrophins and suggested a key role for this helix in channel function [3,4,82]. These studies predicted that various residues between positions 78–87 contribute to anion selectivity, while V78 and F80 were predicted to “be situated close to the permeant ion.” Indeed, a permeant ion must pass this tight restriction to cross the lipid bilayer as later revealed by the channel structures.

### The Neck is a Ca^2^+ -dependent gate for Cl^−^

The initial X-ray structures of cBest1, obtained from two preparations, each using a different detergent, revealed a modest degree of dilation within the neck, specifically at F80 and F84, and this was suggested to represent distinct gating states. The conserved hydrophobic residues within the neck form the narrowest points along the ion conduction pathway and it was proposed that a dehydrated chloride ion may pass with minimal dilation by making anion-pi interactions with the conserved phenylalanines. Subsequent studies determined that the neck must dilate to a greater degree to allow passage of anions and that this X-ray structure of cBest1 must represent a closed state. Mutation of the three neck residues to alanines revealed that the neck is a key regulator of Ca^2+^-dependent Cl^−^ conduction [75,78]. hBest1, cBest1, or bBest2 with the triple mutations I76A/F80A/F84A, deemed “3A” mutant, is constitutively active in the absence of Ca^2+^ [75,78,81]. The 3A mutants conduct Cl^−^ in the absence of Ca^2+^, supporting the hypothesis that the neck poses a physical obstacle to the flow of anions under Ca^2+^ -free conditions. Structurally, the 3A mutants are expected to mimic a conformational state in which the neck residues are pointing away from the central axis, dilating the neck constriction. The X-ray structure of the cBest1 3A mutant (Figure 2a, center) reveals an average neck diameter just large enough (~4.5 Å radius) to allow passage of a 3.3 Å radius hydrated chloride ion (Figure 2c, dotted line), yet the pore-lining neck helix (TMD2) has not undergone a conformational change associated with gating [78]. Consistently, a separate study using molecular dynamics simulations supports the hypothesis that the neck must undergo substantial dilation to pass hydrated ions [83]. Thus, there is a general consensus that there must be a substantial conformational change among the hydrophobic residues of the neck for the bestrophin channel to pass anions.

### The cBest1 open neck

The first open-state, or activated, cryoEM structure of the cBest1 channel was obtained by truncating an autoinhibitory domain in the ordered region of the C-terminal (discussed further in the *Molecular mechanisms of Ca^2+^ -dependent gating* section) [76]. Truncation of cBest1 at residue 345 (cBest1_del345) results in a mutant construct which totally lacks inactivation, proving an excellent specimen to obtain an activated cBest1 structure. In the presence of Ca^2+^, approximately 15% of cBest1_del345 channels are in the open state as shown by 3D classification. In the open-state structure, the pore-lining helix, S2a, which contains residues 79–98, is rotated ~15 degrees, and the three conserved hydrophobic residues no longer point to the channel axis (Figure 2a, right). With this conformational change, the first helical turn of the helix unravels, removing I76 from the channel axis, and the hydrophobic F80 and F84 switch from the most commonly observed rotamer for phenylalanine in the closed position where they point directly into the channel axis, to the second most commonly observed rotameric conformation where they make new hydrophobic contacts and point away from the channel axis. The gating mechanism through the rotation of the neck helix is facilitated by two “tethers,” one above the neck and the other below the neck, as well as a conserved proline (P77) within the helix which couples this rotation with a partial helical unraveling [76]. This gating mechanism dilates the neck from a diameter of <3.5 Å to a diameter of ~13 Å (Figure 2c, solid black line v. gray line, respectively), which is more than enough to accommodate a hydrated chloride ion (solid blue line) and excess water molecules or other larger anions. This conformational change also exposes hydrophilic S79 and G83 of the pore-lining helix to the ion conduction pathway. Thus, the gating of the neck consists of a Ca^2+^ -dependent conformational change of the pore-lining helix TMD2 in which the conserved hydrophobic residues occluding the ion conduction pathway rotate away from the axis and expose a wide and hydrophilic ion conduction pathway.

To date, there is no open state structure for a wild-type bestrophin. All bestrophin open state structures utilized a non-inactivating construct or a mutation that results in a constitutively open channel. It is unclear why all Ca^2+^ -bound bestrophin structures retaining the C-terminal auto-inhibitory domain are in the inactivated state (with the C-terminal tip bound to its allosteric site). The channel does not exhibit fast inactivation in whole-cell patch clamp, as inactivation occurs over the course of minutes. Thus, there may be unidentified factors that modulate channel activity. It is also possible that the protein preparation methodology used for structural studies (extraction with mild detergent with or without reconstitution in amphipathic polymers) does not faithfully represent native proteins in the lipid bilayer. Two potentially important but missing factors from these experimental systems are lipids, which have not been identified in any of the published structures, and post-translational modifications. For example, the phosphorylation of serine 358 has been shown to modulate channel activity by attenuating inactivation (discussed in the “C-terminal dependent inactivation” section) [77,84]. Future studies may need to incorporate these or other factors to recapitulate the native gating process of a wild-type bestrophin channels.

## The neck contributes to anion selectivity

Early experiments guided by sequence alignment support the hypothesis that the second predicted transmembrane domain (TMD2) of hBest1 lines the ion conduction pathway [8]. By analyzing the predicted hydropathy coupled with experiments utilizing charged sulfhydryl-reactive reagents, which are used to introduce a charge at extracellularly exposed cysteine residues, it was correctly determined that TMD2 lines an aqueous solvent-accessible region of the channel. This region corresponds to the conserved neck of KpBest, cBest1, and bBest2. Multiple lines of evidence, including mutagenesis and sulfhydryl reactivity labeling experiments coupled with patch clamp, support the hypothesis that TMD2 plays a role in ion selectivity in mBest2 [3,4,82]. It is also shown that mutating F80 of the neck to a charged residue (arginine or glutamic acid) alters the channel rectification properties and the direction of this rectification is dependent on the charge introduced [4]. Taken further, introducing a negative charge at the analogous residue in dBest1 (from *Drosophila melanogaster*) or by changing the charge with sulfhydryl-reactive reagents can alter the channel selectivity properties. For example, the phenylalanine to glutamine mutation increases cation permeability and the phenylalanine to cysteine mutation exhibits a shift of ionic selectivity after treatment with a negatively charged sulfhydryl reagent. The introduction of a positively charged sulfhydryl reagent into the mutant results in a small shift in the opposite direction. These experiments support the hypothesis that the highly conserved TMD2 contributes to the anion selectivity properties of bestrophins and these experiments are further reviewed in more detail in Hartzell et al. [23]. It is interesting to note that single point mutations within this region generally do not completely obliterate selectivity properties or totally reverse the anion/cation permeability ratio for mBest2 in the described experiments. This observation is in line with the selectivity properties observed in other anion channels, such as CLC and CFTR, where multiple residues are collectively responsible for channel selectivity properties [85].

In line with these previous studies identifying TMD2 as a crucial feature in establishing bestrophin anion selectivity, mutation of specific neck residues can alter the anion/cation permeability ratio in KpBest and hBest1 [72]. KpBest is a Na^+^ channel and also has three hydrophobic residues in the neck, but the middle residue is an isoleucine, rather than phenylalanine. The KpBest neck constriction consists of I62, I66, and F70 (“IIF”), while the analogous neck constriction in eukaryotes is formed by I76, F80, and F84 (“IFF”) (Figure 2b). The selectivity of KpBest can be altered to favor Cl^−^ by the I66F mutation, which turns its IIF neck constriction motif into the eukaryotic IFF motif. Likewise, the F80I mutation in hBest1 decreased the Cl^−^ permeability. The introduction of a positively charged residue in the first neck residue position by the I62R mutation also switches the channel to a Cl^−^ channel rather than a Na^+^ channel. In hBest1, introduction of a negatively charged residue at the analogous location by the I76E mutation flipped the selectivity to favor Na^+^ over Cl^−^. Thus, the charge of the first neck residue in KpBest and hBest1 can tune the channel’s selectivity, and the hydrophobic residue at the middle/second residue location has similar effects. Overall, these results support the hypothesis that the chemical and physical properties of the residues within the neck and within TMD2 contribute to the selectivity properties of KpBest and hBest1. These results underscore subtle differences between KpBest, hBest1, and mBest2, indicating that alternative mechanisms of selectivity may be at play for different bestrophin species and paralogs.

## The aperture: A paralog-specific constriction within the cytosol

**Figure 3.**
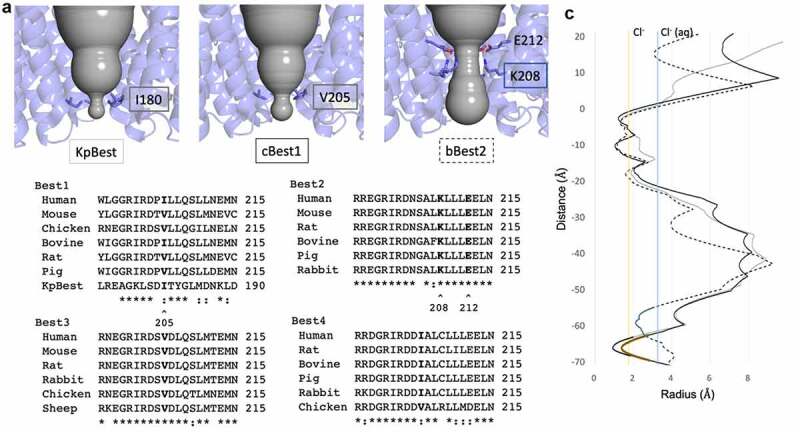
The aperture is a paralog-specific feature. A) Magnified view of the ion conduction pathway at the level of the aperture constriction with one protomer removed. The aperture forming residues are labeled and shown as sticks. B) Multiple sequence alignment for each bestrophin paralog with the aperture constricting residue in bold. Best1 sequence similarity notation does not account for KpBest. “*” denotes fully conserved residue, while “:” denotes similar residue C) HOLE diagram for KpBest (gray line), cBest1 (solid black line), and bBest2 (dotted black line) with aperture-forming residue shown in color corresponding to their label in A. Vertical solid yellow line indicates the radius of dehydrated Cl^−^, while solid blue line indicates the radius of hydrated Cl^−^

The cytosolic vestibule is characteristic of the three reported bestrophin structures [72,74,81]. The ion conduction pathway extends from the membrane ~45 Å into the cytosol within the enclosed vestibule of the cytosolic domain. This region of the ion conduction pathway varies in diameter, starting at approximately 8 Å just below the neck, widening to approximately 16 Å at its widest point, and narrowing again at the cytosolic entry to the channel (Figure 3c). The narrowest constriction at this point is deemed the “aperture” and represents a key feature of bestrophin channels. Importantly, multiple sequence alignment of the aperture region reveals key differences between the paralogs (Figure 3b).

**Figure 4.**
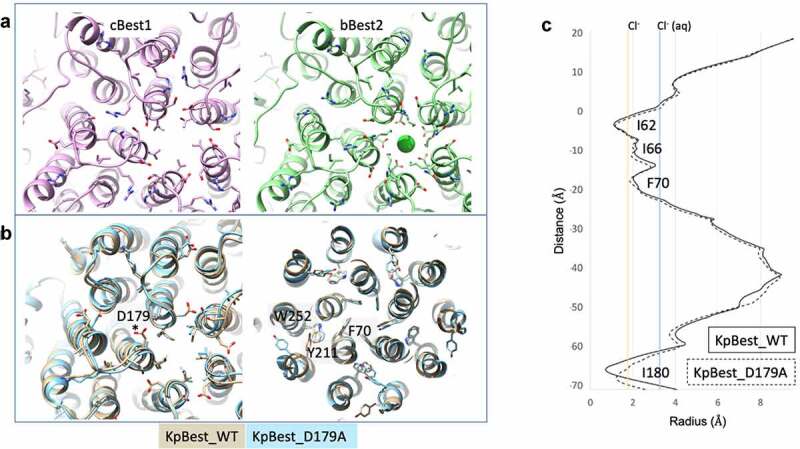
A) Aperture of eukaryotic bestrophins, cBest1 on the left and bBest2 on the right. B) Overlay of the aperture of KpBest (gold) with the D179A mutant (blue) on the left, demonstrating the small conformational change that accommodate modest dilation of the aperture. On the right, small conformational changes within the gating apparatus of the neck observed with the D179A mutation. C) HOLE diagram depicting the small dilation of the aperture in KpBest with D179A mutation

The narrowest point of the cytosolic aperture is formed by I180 in KpBest and by V205 in cBest1 (Figure 3a, left and center, respectively) [72,74,75,78]. Multiple sequence alignment reveals that Best −1, −3, and −4 have a hydrophobic valine or isoleucine at position 205 (Figure 3b), which forms the tightest local constriction. The KpBest and cBest1 homologs serve as prototypes for an aperture containing the bulky isoleucine and smaller valines in the aperture, respectively. On the other hand, the position 205 in Best2 is not fully conserved and can be a serine or glycine (Figure 3b) [81]. In bBest2, a conserved lysine (K208) and conserved glutamic acid (E212) together form a salt bridge that comprises the aperture, where a putative chloride ion is bound in the center of the ion conduction pathway in the cryoEM structure (Figure 3a, right and Figure 4a, right). This feature of bBest2 is required for its Ca^2+^ -dependent activity, as the E212A mutant is gating-deficient (Cl^−^ current does not increase in the presence of stimulating [Ca^2+^]) [81]. As a result, the aperture can be composed of hydrophobic residues, as in Best -1, -3, and -4, or hydrophilic residues, as in Best2. Studies have identified several key features of the aperture, including contributions to Ca^2+^ -dependent gating and anion selectivity.

### Functional and structural evidence for aperture gating

In addition to Ca^2+^ -dependent gating of the neck, evidence supports the hypothesis that the aperture can also gate to facilitate anion flow across the membrane. In the case when isoleucine forms the aperture, as in hBest1 and KpBest, the constriction with radius of 0.9 Å is too tight to that allow the passage of a dehydrated Cl^−^ ion, which has a radius of 1.8 Å (Figure 3c, solid gray line) [75]. In the case when valine forms the aperture, as in cBest1, the constriction has a radius of 1.3 Å (Figure 3c, black line) and may be just wide enough to allow passage of dehydrated Cl^−^ (Figure 3c, yellow line) when accompanied by a small dilation [74,76,78]. As the size of the permeating anion increases (for example, for Br^−^ with radius 2.0 Å or for I^−^ with radius 2.2 Å), a larger dilation must occur to facilitate passage of the ion through the aperture.

Consistent with the aperture’s size restraint on permeating ions, the I180A aperture mutation in KpBest results in channels with increased single-channel opening probability. Similarly, the analogous mutation in hBest1, I205A, results in significantly larger whole-cell currents [72]. Thus, because the aperture constriction is too small to allow passage of dehydrated ions without substantial dilation, a complete gating model must include dilation of both the aperture and the neck to account for an anion’s passage through the entire length of the channel.

The aperture of KpBest undertakes a small asymmetric dilation when modeling the D203A human Best1 gain-of-function mutation (D179A in KpBest) [75]. D179 of KpBest is located on a conserved intracellular loop adjacent to I180, which forms the aperture, and makes a salt bridge interaction with R172. Although this mutation induces a modest conformational change (Figure 4b), it opens the aperture from a radius of 0.9 Å to 1.5 Å (Figure 4c), representing a substantial dilation to begin accompanying the passage of a dehydrated Cl^−^ ion (Figure 4c, yellow line). Coupled with “thermal breathing,” this conformational change could account for anion passage through this constriction, although it is not yet apparent how Ca^2+^ binding near the membrane translates to movements at the aperture.

### Gating of larger anions

**Figure 5.**
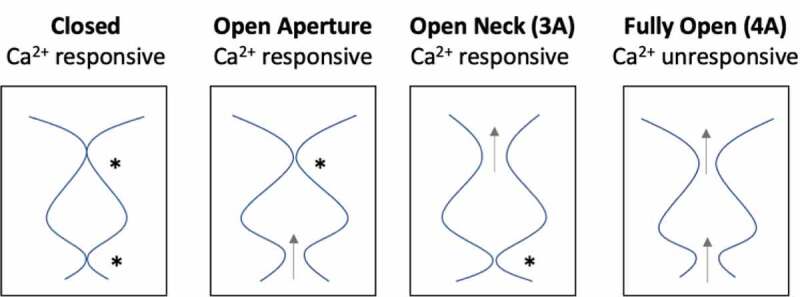
Schematic diagram depicting functional effects of open aperture, open neck (3A), and fully open (4A) mutants. “*” depict Ca^2+^-responsive gates of each mutant, while gray arrows depict a dilated constriction that allows ion flow in the absence of Ca^2+^

Bestrophin channels pass the large anions thiocyanate (SCN^−^) and methanesulfonate (Mes^−^), which have dehydrated radii of 2.2 Å and 2.6 Å, respectively. For these larger anions to pass through the aperture, there must be a substantial dilation from a radius of 0.9 Å (for I180 in KpBest or the predicted I205 in hBest1) or 1.3 Å (for V205 in cBest1). Ca^2+^ -independent currents are also observed with Mes^−^ as the predominating anion in solution for the aperture-only mutant I205A, the neck-only mutant 3A, and the combined aperture–neck- mutant 4A, indicating that both the neck and aperture exhibit spontaneous opening [75]. Furthermore, Ca^2+^ stimulates the current in the 3A mutant and the I205A mutant, but not in the 4A mutant, indicating that each gate is at play for large anions and the 4A mutant resembles a fully “open” channel capable of conducting this large anion (Figure 5).

In contrast to the Best -1, -3, and -4 paralogs, Best2 has an aperture constriction that is large enough to allow passage of a dehydrated Cl^−^ ion without any dilation [81]. Nevertheless, bBest2 still passes the large anions I^−^ and Mes^−^, which are too large to pass without dilation. The K208A and 3A mutants also increase conductance with the addition of stimulating [Ca^2+^] when Cl^−^ is replaced by I^–^ in the buffer, indicating that both gates are at play for passage of this larger ion with radius 2.2 Å. Thus, the aperture of bBest2 is a Ca^2+^ -dependent gate for larger anions, but not for smaller anions.

### *The* trans *promotive effect of methanesulfonate*

Additionally, Mes^−^ induces a “*trans*-promotive effect” on hBest1 and bBest2, significantly stimulating the current when the large ion is exposed to the extracellular side of the membrane [75,81]. This *trans* promotive effect increases Cl^−^ efflux (inward current), as well as Mes^−^ influx (outward current) to the cell. Thus, Mes^−^ in the extracellular buffer promotes Cl^−^ outward movement in *trans*. This *trans* promotive effect was abolished in the hBest1 and bBest2 3A neck mutants, but retained in the aperture mutants, indicating that the effect likely acts by dilating the neck [75,81]. Furthermore, the K208A mutant conducts similar Mes^−^ currents with or without Ca^2+^, indicating that the aperture is the only gate impeding Mes^−^.

### The aperture contributes to anion selectivity in the cBest1 channel

Results from cBest1 suggest that V205, which forms its tightest constriction within the aperture, is responsible for maintaining the channel’s lyotropic permeability sequence [78]. When V205 is mutated to the smaller alanine, the channel no longer maintains a lyotropic sequence of relative anion permeability (SCN^−^ > NO_3_^−^ > I^−^ > Br^−^ > Cl^−^). These results support the hypothesis that the size of the aperture dilation dictates the sequence of relative permeability for different anions, such that an anion must become at least partially dehydrated to pass V205. This model presents a mechanism in which cBest1 has one gate (the neck) and one selectivity filter (the aperture), yet whether these results translate to other bestrophins with an isoleucine at positions 205, such as hBest1, has yet to be shown.

## Molecular mechanisms of Ca^2+^ -dependent gating

### The Ca2+ Clasp: A highly conserved Ca^2^+ binding site

Bestrophins respond to free [Ca^2+^] in the nanomolar concentration range; thus, they are expected to have a conserved Ca^2+^ binding site to explain such exquisite sensitivity. It is important to note that KpBest is not Ca^2+^ sensitive and lacks this binding site [72]. Initial functional studies on hBest1 informed by multiple sequence alignment identified a cluster of conserved acidic residues between position 293–308 that resemble the “Ca^2+^ bowl” of BK channels [23,86–88]. These residues were shown to be required for Ca^2+^ dependent activity as their mutation resulted in Ca^2+^ -insensitive channels [88]. Additional functional studies underscored the importance of conserved acidic residues and those involved in Ca^2+^ binding within this region for Ca^2+^ -dependent function, including implications for Best disease patient mutations [89,90,23,75,91,7,78,88,92–94].

**Figure 6.**
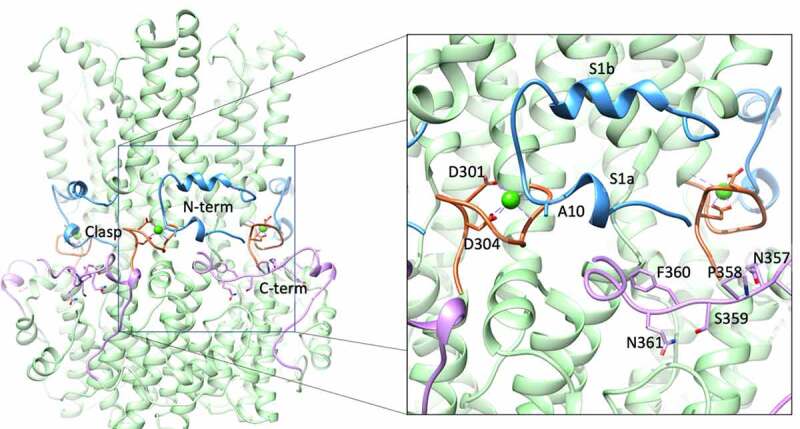
The Ca^2+^ -sensing apparatus of bBest2 with magnification on the right. The N-terminal extension (residues 2–27) is blue, the Ca^2+^ -clasp (residues 295–306) is orange with Ca^2+^ coordinating acidic residues depicted as sticks, and the C-terminal extension (340–367) is pink with residues of the c-terminal inactivation region depicted as sticks

The initial X-ray structure of cBest1 in the presence of 5 mM CaCl_2_ captured the channel in a Ca^2+^-bound state, revealing five Ca^2+^ binding sites per channel (one per protomer) [74]. This X-ray structure is nearly identical (R.M.S.D = 0.71 Å) to the bBest2 cryoEM structure solved in the presence of high Ca^2+^ (Figure 6) [81]. Each Ca^2+^ ion is coordinated with pentagonal bipyramidal geometry and is directly coordinated by the acidic side chains of the conserved D301 and D304, as well as the carbonyl oxygen atoms of A10, Q293, and N296, and one water molecule. The conserved acidic residues of the clasp that do not coordinate Ca^2+^ (E300, D302, and D303) likely increase the local concentration of Ca^2+^ and may aid in its recruitment or the recruitment of the Ca^2+^ -dependent N- and C-termini. A single water molecule is bound to Ca^2+^ and coordinated by the carbonyl oxygen atoms of V9 and E292. This water molecule was originally identified in the cBest1 X-ray crystal structure and is also present in the bBest2 cryoEM structure, underscoring its importance in maintaining the Ca^2+^ binding geometry for both cBest1, bBest2, and likely other eukaryotic bestrophins [74,81].

### Ca^2^+ -dependent ordering

Recent breakthroughs in cryoEM have enabled the relatively high-throughput study of protein structure under varying buffer conditions. The first cBest1 X-ray structures were all solved in the presence of 5 mM Ca^2+^ and it was predicted that subsequent Ca^2+^ -free structures would reveal dramatic conformational changes [74]. On the contrary, recent cryoEM studies revealed a remarkable similarity between the Ca^2+^ -bound and Ca^2+^ -free structures with key differences in three regions of the protein: the N- and C-terminal extensions and the Ca^2+^ binding site [76,81].

In all high Ca^2+^ structures, the N-terminal extension, which comprises residues 2–23 and containing helices s1a and s1b (Figure 6, blue), is fully ordered and wraps around the adjacent protomer with the carbonyl oxygen atom of A10 making contact with the adjacent protomer’s Ca^2+^ ion [74,76,81]. This interaction serves as an anchor to stabilize the five N-terminal extensions as they constrict the periphery of the channel like a belt, just below the transmembrane domain. The C-terminal extension is also ordered, wrapping across the adjacent protomer and toward the Ca^2+^ site of the next protomer over (Figure 6, pink). In the Ca^2+^ -free cryoEM condition for cBest1, both the N- and C-terminal extensions are disordered [76]. In contrast, the Ca^2+^-free cryoEM structures of bBest2 reveal that these regions can be ordered or disordered in the absence of Ca^2+^ [81]. Thus, bBest2 is capable of N- and C-terminal extension ordering in the absence of Ca^2+^, although the functional implications of Ca^2+^ -independent N- and C-termini ordering are not clear. Ordering of the N-terminal extension also tethers TM1, helping stabilize TM2.

### C-terminal dependent inactivation

The different bestrophin channel paralogs and homologs can have widely varying characteristics, including major differences in their sensitivity for [Ca^2+^], whole-cell current amplitude upon heterologous expression, and differences in activation and inactivation kinetics. It was initially reported that bestrophin current “ramps up” over the course of many seconds to minutes, depending on the bestrophin paralog or species, in response to stimulating [Ca^2+^] and that this current “runs down” or slowly diminishes over time [2,6,77,82,88]. The current rundown is both time- and [Ca^2+^]- dependent and can be considered a time- and concentration-dependent inactivation. Tsunenari et al. [8],first showed that mBest3 and hBest4 produce small Cl^−^ currents upon heterologous expression when patched with standard conditions [8]. In fact, significant currents were only obtained with large amounts of transfecting cDNA and very long (4 s) and large (100 mV) hyperpolarizations [2,8]. Through a series of truncations followed by more precise mutation of individual residues, an auto-inhibitory region was identified within the C-terminus (deemed ^356^IPSFLGSTI^364^, Figure 6 pink, labeled residues) that, when removed or mutated, results in large Ca^2+^ -dependent currents under more physiological patch clamp conditions [2]. This auto-inhibitory domain shares the consensus sequence SFXGS in all vertebrates and the first serine (S358) within this site of hBest1 is the target of phosphorylation by PKC or PKA, which attenuates inactivation [2,6,21,77,84,88,95]. The phosphorylation of S358 was also found to mediate a direct interaction between hBest1 and its binding partner 14-3-3 γ to increase the Best1 surface expression and activity in cultured astrocytes [96]. Thus, the presence of this conserved motif on the C-terminus and the phosphorylation state of S358 regulates inactivation.

It is interesting to note that bestrophins may be differentially regulated by phosphorylation, as evidenced by the apparently disparate kinase substrate motifs present within hBest1 and dBest1 [2,6,88,97]. Importantly, dBest1 activity was similarly increased by kinase activity and decreased by phosphatase activity, yet dBest1 lacks the PKC/PKA phosphorylation site (S358) and was shown to be a substrate for CaMKII. Thus, while S358 is important for regulation of mammalian bestrophins, other sites may be important, and there may be species- or paralog-specific differences in regulation.

The autoinhibitory domain on the c-terminal inactivates the channel by binding to an allosteric site that directly modulates channel inactivation by inducing closure of the neck [76,77]. The initial Ca^2+^ -bound X-ray structure of cBest1 in which the C-terminal autoinhibitory domain is bound was in an inactivated state [74]. Interestingly, the crystallization chaperone (a Fab monoclonal antibody fragment), which was required for the growth of well-diffracting crystals suited for X-ray structural studies, was bound on the periphery of the cytosolic domain, where it prevented unbinding and disinhibition of the autoinhibitory domain. This biochemical information proved instrumental in obtaining an open-state bestrophin structure [76].

### ATP facilitates Ca^2^+ -dependent activation

In line with the potential need for other factors to stimulate channel opening, ATP potentiates Ca^2+^ -dependent activation of hBest1 and directly activates KpBest [79]. This direct interaction is stronger for the non-hydrolyzable ATP analog ATP-y-S (Kd = 16 μM) than for ATP (Kd = 254 μM), while ADP made much weaker interaction (Kd = 1.3 μM) and AMP made no measurable interaction. ATP significantly increases KpBest channel opening probability in a dose-dependent manner in bilayer experiments using purified KpBest. Multiple lines of evidence support the hypothesis that hBest1 exhibits ATP-dependent potentiation of Ca^2+^ dependent Cl^−^ currents in RPE cells. Using RPE cells derived from human induced pluripotent stem cells (iPSC-RPE), which express hBest1, it was shown that Ca^2+^ -dependent Cl^−^ currents are potentiated by ATP and non-hydrolyzable ATP-y-S, indicating that the potentiation is not phosphorylation dependent. These currents were blocked by the nonspecific Cl^−^ channel blocker niflumic acid (NFA) and are dependent on intracellular Ca^2+^, as ATP alone does not stimulate Cl^−^ currents. Notably, iPSC-RPE Ca^2+^ -dependent Cl^−^ currents are approximately 3x larger in the presence of 10 mM ATP + 0.6 μM Ca^2+^ than with 0.6 μM Ca^2+^ alone, underscoring the potential physiological ramifications of this channel feature. Currents remained robust with the substitution of ATP by ATP-y-S (~2.5x higher than currents with Ca^2+^ alone), indicating that phosphorylation was not the major mechanism of channel potentiation. Similarly, bBest2-mediated Ca^2+^ -dependent Cl^−^ activity was enhanced by ATP (Kd = 560 μM) and ATP-y-S (Kd = 10 μM) upon heterologous expression in HEK293 cells, while bBest2 directly interacts with ATP and ATP-y-S in microscale thermophoresis experiments, suggesting that ATP-dependent potentiation is a conserved feature of bestrophins [79]. Although there is no ATP-bound bestrophin structure, the putative ATP binding site was functionally mapped to a conserved motif on “Loop 2,” which sits between the helix S2h and S3a of eukaryotic bestrophins, directly adjacent to the aperture, an ideal location to modulate the aperture gate. Moreover, a patient mutation in this loop disrupts ATP binding and completely abolishes ATP-dependent potentiation of Ca^2+^ -dependent Cl^−^ activity in patient-derived iPSC-RPE [79].

### One-gate (neck in Best2) vs. dual-gate (neck and aperture Best1)

Electrophysiology experiments on bestrophin mutants demonstrate that both the aperture and neck gate are in response to Ca^2+^ [31,72,75,81]. When one gate is mutated to alanine, which results in a dilated constriction such that a dehydrated Cl^−^ may pass, and the other gate is left intact, the channel still responds to increasing Ca^2+^ with increased current (Figure 5). hBest1 3A (open neck) and I205A (open aperture) mutants both exhibit modest Ca^2+^ -independent activity, but the currents increase with the addition of Ca^2+^, suggesting that these mutants are “leaky” and not quite fully open. Thus, even when one of these gates is opened by the alanine mutation, there is still a substantial increase upon Ca^2+^ addition, indicating that gating still occurs at the non-mutated gate (Figure 5).

The high level of sequence conservation within each bestrophin paralog suggests that each one fulfills a different and conserved function. The dual gating mechanism of hBest1, in which both the aperture and neck must dilate to accommodate a passing Cl^−^ ion, differs substantially from that of bBest2, in which the aperture can accommodate a passing Cl^−^ ion without dilation [75,81]. Thus, for smaller ions that are present in physiological systems (i.e., excluding Mes^−^ and SCN^−^), bBest2 has one gate and hBest1 has a dual-gate mechanism. This difference is in accord with the observation that Best2 conducts HCO_3_^−^, which is a bigger anion compared to Cl^−^, in colonic goblet cells [55].

### Patient mutations reveal gating mechanisms

Elucidation of mutant Best1 pathological mechanisms is facilitated by a more thorough understanding of the Best1 structure–function relationship. There are over 250 disease-causing *BEST1* mutations, with over 120 mutations causing amino acid substitutions. Many of these missense mutations reside in key regions that contribute to the protein function, such as the neck-forming TM2, the Ca^2+^-clasp, and regions that may be important for protein stability [98]. A study using KpBest elucidated structural mechanisms of two Best1 disease-causing mutations and revealed that long-range structural perturbations within the TM can result from mutation of a single residue near the aperture (Figure 4c) [31]. A separate study used KpBest to elucidate the structural and functional effects of three patient-derived gain-of-function mutations, including one mutation that opens the aperture gate (I205T), one that alters gating at the neck (Y236C), and a third (D203A) that alters the link between the neck and aperture gates [75].

## Multiple regions contribute to anion selectivity

Early studies determined that bestrophins predominantly pass monovalent anions and that their relative permeability follows the sequence SCN^−^ > NO_3_^−^ > I^−^ > Br^−^ > Cl^−^ > F^−^, which corresponds to the inverse of the ions’ dehydration energies [3,4,5,7,54,60,74,81,78,88,99]. That is, the permeability sequence is lyotropic, and the more easily an anion is dehydrated, the more easily it passes through the channel, presumably through a process that is limited by the thermodynamics underlying dehydration of the anion [23,67]. Various functional studies and more recent structural studies have shed considerable insight into the chemical and structural features that give rise to the bestrophin channel’s anion selectivity properties. Different bestrophin paralogs and species exhibit widely varying individual channel properties (Table 1) with specific regions identified as contributors to anion selectivity. It is likely that the pore-lining residues of the neck (TMD2), the aperture, charged residues lining the ion conduction pathway, and the multiple chloride binding sites within the channel collectively contribute to the anion selectivity of bestrophin channels. It is possible that anion permeability can be tuned on an individual channel basis based on the presence and variability of these features. It is also possible that more channel characteristics contributing to anion selectivity are yet to be uncovered.

### Chloride binding sites present within the cBest1 channel

Anomalous scattering was used to identify ions bound to the cBest1 X-ray structure [74]. In addition to the Ca^2+^ ions within the acidic clasp, one K^+^ ion and three Cl^−^ ions were bound per protomer. The bound Cl^−^ ions are located near the ion conduction pathway, such as inside the cytosolic vestibule, or above the neck on the extracellular side, with a total of 15 Cl^−^ ions bound per channel. They are accessible to the solvent within the permeation pathway, and each Cl^−^ ion is bound to a backbone amino nitrogen at the site of a break in the alpha-helix. These Cl^−^ binding sites are absent in KpBest, which is a cation channel, and have been proposed to contribute to anion conduction by increasing local anion concentrations [78]. Interestingly, there is no corresponding density for 2 out of 3 of these Cl^−^ sites in the bBest2 cryoEM maps, which may partially explain the higher sodium conductance in bBest2 when compared to cBest1. Thus, it is likely that these internal Cl^−^ binding sites along the pore contribute to the channel’s relative anion/cation selectivity [74].

### The Aperture and other pore-lining residues contribute to anion selectivity in the bBest2 channel

In contrast to the other bestrophin paralogs (1/3/4), Best2 does not have a conserved hydrophobic residue within the aperture. The aperture of bBest2 is formed by a conserved lysine (K208) and conserved glutamic acid (E212), which interacts to form a salt bridge [81]. In the bBest2 structure under high Ca^2+^, low Ca^2+^, and Ca^2+^ -free conditions, this salt bridge was maintained and always has a bound ion, which comes into close contact with the terminal nitrogen of K208, sitting along the axis of symmetry. Functional analysis of the K208A mutant indicated that this residue contributes to the anion selectivity of the channel as K208A mutant channels had decreased relative permeability for the larger anions I^−^. Importantly, the relative permeabilities of other anions was not altered in the K208A mutant, underscoring a key difference from cBest1, where the V205A mutation completely abolished the lyotropic permeability sequence [78,81].

In addition to the aperture, two conserved basic residues along the ion conduction pathway of bBest2 were identified to contribute to the anion selectivity [81]. A conserved lysine, K265, at the extracellular entrance to the channel is in a position to interact with a passing anion, and the K265A mutant exhibits reduced selectivity for I^−^ and SCN^−^ in bBest2; H91, located directly beneath the hydrophobic neck of bBest2, is conserved in all Best2 species analyzed except for hBest2, where it is an asparagine, and was determined to be critical to maintain the selectivity for SCN^−^. Thus, in addition to the internal chloride binding sites, the aperture, and TMD2, the charged residues that make local constrictions along the ion conduction pathway contribute to the channel’s particular anion selectivity properties.

## Therapy opportunities and future directions

Significant progress in the development of therapeutics to treat bestrophinopathies has been made in recent years. Induced pluripotent stem cell (iPSC) technologies utilizing Best disease patient-derived tissue and knock-in patient mutations have facilitated insights into the molecular mechanisms of VMD2 mutations and helped reconcile these with clinical observations. To this end, the established protocol to differentiate iPSCs to an RPE phenotype has been instrumental in enabling genetic, functional, and pharmacological analyses of Best disease [100–107]. In particular, human iPSC technologies allow for relatively high-throughput analysis of disease-causing mutations without the need for slow and potentially irrelevant rodent models (*BEST1*-KO mice do not have any retinal abnormalities) [33,34]. Over 250 disease-causing mutations have been identified in the VMD2 gene, and iPSC-RPE-based models have been used to identify a wide array of cellular dysfunctions in patient-derived tissues with increased throughput. The wide range of dysfunctions induced by Best1 disease-causing mutations include a reduced ability of the RPE to efficiently phagocytose or process photoreceptor outer segment, changes in intracellular Ca^2+^ handling, altered fluid flux, reduced Best1 protein expression, ER quality control-dependent and -independent mechanisms, increased or decreased anion permeability, as well as direct effects to the channel’s CaCC activity [31,108,109–114]. These models have enabled diverse therapeutic strategies, which include the development of pharmacological chaperones to restore Best1 expression, utilizing CRISPR/Cas9 to repair mutant *BEST1* genes, and a curcumin-based nanomedicine approach to increase Best1 mRNA and protein levels [109,113,115,116]. Notably, loss of Best1 function in patient-derived iPSC-RPE cells can be fully rescued by AAV2-mediated WT *BEST1* gene augmentation [117], while gain-of-function *BEST1* mutations are also rescuable by a combination of gene augmentation with CRISPR/Cas9-mediated knockdown of endogenous Best1 expression [118], underlying a bright future of stem cell- and/or gene-based therapies for the treatment of bestrophinopathies.

The retina presents itself as an ideal candidate for experimental stem cell- and gene therapy-based strategies for multiple reasons. The relative accessibility of the retina to surgery and noninvasive monitoring techniques enables routine intervention and quantitative analysis of disease outcomes, the blood-retina barrier provides immune privilege, and complications are rarely life threatening [98]. A recent study demonstrated a beneficial safety profile and satisfactory efficacy for AAV2-mediated delivery of the *BEST1* gene to reverse key ultrastructural and molecular Best disease characteristics in a naturally occurring canine Best disease model [119]. Autologous transplantation of patient-derived lab-grown iPSC-RPE holds the potential to fully restore RPE function in bestrophinopathy patients, and multiple studies utilizing various types of cell-based therapies are underway to repair degenerative retinal conditions [19,120]. Despite the diverse repertoire of disease-causing mutations and pathologies that lead to degenerative retinal disorders, a general therapeutic mechanism for directly replacing degenerative tissue or supplementing degenerating tissue with cell-derived soluble factors holds promise.

Treatment of bestrophinopathies and the continued investigation of the role of the various bestrophin paralogs in disease and physiology will benefit from detailed mechanistic insights into the structure–function relationship of bestrophins. A better understanding of disease mechanism benefits from biophysical, molecular, and genetic studies, and can help reveal potential routes of therapeutic intervention, especially through structure-based drug design. Future molecular studies on bestrophins may focus on developing paralog-specific inhibitors or activators. While no direct channel activators have been identified, the cytosolic ATP binding site presents an enticing prospect for the development of therapeutic Best1 channel modulators. On the other hand, Best2-specific inhibitors may provide utility in altering aqueous humor dynamics with the end goal of lowering intraocular pressure to treat glaucoma. Future studies may also seek to identify paralog-specific structural differences that give rise to the distinct biophysical features of each paralog. To date, the structure–function studies on three bestrophin homologs, KpBest, cBest1, and bBest2 have shed considerable insight into their mechanisms of function and reveal distinct mechanisms of gating. These studies also lay the foundational groundwork to begin pharmacological development by rational drug design and will guide future biophysical analyses in elucidating their function in physiology and disease.
